# The Mining Minds digital health and wellness framework

**DOI:** 10.1186/s12938-016-0179-9

**Published:** 2016-07-15

**Authors:** Oresti Banos, Muhammad Bilal Amin, Wajahat Ali Khan, Muhammad Afzal, Maqbool Hussain, Byeong Ho Kang, Sungyong Lee

**Affiliations:** 1 Department of Computer Engineering, Kyung Hee University, 1732 Deokyoungdae-ro, Giheung-ug, Yongin-si, 446-701 Korea; 2 School of Computing and Information Systems, University of Tasmania, Churchill Avenue Hobart, Tasmania, 7005 Australia

**Keywords:** Human behavior, Digital health, dHealth framework, Quantified self, Wearable sensors, Big data, Cloud computing, Context-awareness, Knowledge bases, User experience

## Abstract

**Background:**

The provision of health and wellness care is undergoing an enormous transformation. A key element of this revolution consists in prioritizing prevention and proactivity based on the analysis of people’s conducts and the empowerment of individuals in their self-management. Digital technologies are unquestionably destined to be the main engine of this change, with an increasing number of domain-specific applications and devices commercialized every year; however, there is an apparent lack of frameworks capable of orchestrating and intelligently leveraging, all the data, information and knowledge generated through these systems.

**Methods:**

This work presents Mining Minds, a novel framework that builds on the core ideas of the digital health and wellness paradigms to enable the provision of personalized support. Mining Minds embraces some of the most prominent digital technologies, ranging from Big Data and Cloud Computing to Wearables and Internet of Things, as well as modern concepts and methods, such as context-awareness, knowledge bases or analytics, to holistically and continuously investigate on people’s lifestyles and provide a variety of smart coaching and support services.

**Results:**

This paper comprehensively describes the efficient and rational combination and interoperation of these technologies and methods through Mining Minds, while meeting the essential requirements posed by a framework for personalized health and wellness support. Moreover, this work presents a realization of the key architectural components of Mining Minds, as well as various exemplary user applications and expert tools to illustrate some of the potential services supported by the proposed framework.

**Conclusions:**

Mining Minds constitutes an innovative holistic means to inspect human behavior and provide personalized health and wellness support. The principles behind this framework uncover new research ideas and may serve as a reference for similar initiatives.

## Background

Healthcare systems are facing unprecedented financial limitations at a time of rising demand for their services [1]. The magnitude of these constrains makes utterly necessary to change current care models in a bold manner, from late disease management to preventive personalized health, involving a major shift in when, where and how care and support is delivered to each particular patient and service user [2]. In fact, it is generally recognized that most prevalent diseases are partly caused or aggravated by lifestyle choices that people make in their everyday life. Unwholesome diets, tobacco use and sedentary conducts, among other unhealthy habits, potentially contribute to develop severe illnesses [3, 4] and also limit the effectiveness of medical treatments [5]. Thus, enabling people to make healthier choices, to be more resilient, and to deal more effectively with illness and disability when it arises, turns to be a fundamental part of this necessary new health perspective.

Information and communication technology is called upon to be a cornerstone of the new health era, playing a crucial role in empowering people to take charge of their own health and wellness, by providing them timely and ubiquitously with personalized information, support and control [6]. In fact, an extraordinary interest has been lately shown by the industry in the development of specific applications and systems for health and wellness management, particularly boomed by the growth of wearable and mobile technology [7]. The immediate targets of these solutions are healthy lifestyle services, especially oriented to the fitness domain, which primarily allow to track primitive user routines and provide simple motivational instructions. For example, mainstream commercial systems such as Withings Activite [8], Garmin Vivofit [9], Fitbit Surge [10] or Misfit Shine [11], which consist of sensorized bracelets and gadgets normally accompanied by mobile apps, provide some basic healthy recommendations based on the measured taken steps or slept hours. More prominent health and wellness systems have been shown at the research level, for example, to alert on physical conditions [12] or detect chronic illnesses [13], yet most of them are prototypes or work-in-progress. Some of these systems also provide educational modules and personal coaching for promoting healthier lifestyles and managing health conditions [14]. Despite their interest, main limitations of these solutions refer to misperformance, limited scope and lack of interoperability with other similar systems and applications.

To overcome the shortcomings of application-specific solutions and leverage the potential of health information systems in a wide sense, general frameworks capable of managing these resources are required. A few attempts are found in this respect in the literature, for example, in [15] a middleware framework integrating multiple interfaces and multiparameter monitoring of physiological measurement is presented. In [16], distributed signal processing algorithms for the analysis and classification of sensor data are provided as part of a framework for rapid prototyping of body sensor networks. A mobile platform to collect users’ psychological, physiological and activity information for mental health research is presented in [17]. The authors of [18] propose a healthcare platform particularly devised for interfacing and processing data from body-worn physiological sensors and home appliances, with a proven utility in daily medication management. A novel framework that provides advanced functionalities for resource and communication abstraction, wearable health data acquisition and knowledge extraction is introduced in [19]. Most visible initiatives are especially being underpinned in the mobile health domain. That is the case of [20], an open mobile health project to help developers produce digital health data as useful and actionable as possible. Google Fit [21] by Google, SAMI [22] by Samsung or HealthKit [23] by Apple are examples of new commercial platforms also devised to integrate and share users health data among diverse health and wellness applications.

Despite important contributions have been made through these platforms, there is still much room for improvement. For example, most mobile health frameworks are bound to the computational capabilities of the smartphone, require continuous maintenance and updates of end-user applications and normally trap data into their devices. Moreover, multiple systems and applications can generate similar health data and outcomes leading to unnecessary redundancy and overcomputation. These systems mostly operate on-demand, thus determinants of health and wellness states can also be lost if not registered in a continuous manner. Platforms devised to share and integrate health and wellness data underutilize cloud resources while simply using them for storage. In the light of these limitations we present Mining Minds [24, 25], an innovative distributed framework that builds on some of the most prominent digital technologies to enable the provision of personalized healthcare and wellness support. This framework is particularly devised to seamlessly investigate on people’s behavior and lifestyles in an holistic manner through mining human’s daily living data generated through heterogeneous resources. Mining Minds aims to innovatively exploit the potential of cloud computing not only for storage but also for high performance computation supporting the discovery of personal and public health and wellness patterns, of primal necessity to facilitate proactive and preventive support.

## Requirements of a digital health and wellness framework

Diverse types of data are normally required to neatly describe a person’s health and wellness state, ranging from physical-sensory- and logical-personal profile and interests-, to social-human relations- and clinical-medical-data. Many technologies are increasingly available for the collection of these data, such as wearable devices, ambient sensors, social networks or advanced clinical systems. Thus, an important requirement of a digital health and wellness framework is to provide a certain level of abstraction from heterogeneous resources to make their utilization transparent to the user. Health and wellness data go beyond standardized structured formats such as “traditional” electronic health records, particularly including other multimedia and unstructured data. Therefore, another primal requirement is to be capable of dealing with this dimension of heterogeneous data, as well as the underlying implications of the management of structured, semi-structured and unstructured data.

Not only data variety constitutes a key factor, but also data volume. Massive amounts of data are generated over time on and around the subject with the advent of new sensing and multimedia technologies. Accumulating and digesting these amounts of data are not trivial tasks and need to involve sophisticated processing and storage mechanisms to enable the persistence and availability of the data. Similarly, the rapid pace of data generation makes it necessary to also take into account data velocity as a reference factor. This proves to be especially challenging when referred to data that represents real-time regular monitoring, such as continuous electrocardiogram measurements or body motion data. Another important concept that applies to health and wellness data is veracity. Different data types may represent similar concepts or contradict each other, or even be of little interest. Therefore, digital health and wellness frameworks should count on governance mechanisms to determine the consistency of the data, ensuring it is certain, meaningful, clean and precise.

Extracting the determinants of health and wellness is a very challenging task that requires more than simply collecting and persisting personal data. Accordingly, digital health and wellness frameworks must include automatic intelligent mechanisms to process person-centric data and extract interpretable information and insights for ensuring a personalized health and wellness support. Moreover, insights should not only be gained from individual users but from the collectivity. Thus, another important requirement consists in the application of advanced techniques to process information in “de-identified” form to enable population management and deeper insights into cause and effect. These insights can be particularly leveraged by health and wellness care systems to extend, adapt and evolve the knowledge provided by human domain experts.

Health and wellness information and knowledge are principally devoted to support advanced care services. Mechanisms such as alerts, recommendations or guidelines are particularly used as services to catalyze both information and knowledge to be delivered in a human-understandable fashion to users and stakeholders in general. However, most digital health and wellness systems only support general services that do not differentiate among people's particular needs or interests. Therefore, an important requirement is to provide services that operate on a person-centric manner. To do so, expert systems are required, for example, to precisely map user needs to the best possible recommendations, personalize the recommendations explanation or customize the mechanisms for the communication of these recommendations.

Users of health and wellness systems may be of a very diverse nature and play different roles. For example, busy patients may require to get a quick glimpse of their health conditions, fitness enthusiasts wish to observe a detailed description of their vitals and clinical experts be interested in an “in-depth” description of both health and wellness outcomes of multiple people. Accordingly, user interfaces need to be customized to the needs of each particular subject. Similarly, the user experience is of worth consideration. Users perceptions of system aspects such as utility, ease of use and efficiency should be taken into account to provide the most personalized experience. In fact, the user experience is dynamic as it is constantly modified over time due to the person changing circumstances. Thus, user responses and behavior need to be continuously tracked to support a sufficient level of personalization that helps guarantee adoption and engagement.

Finally, as it may be obvious, but unfortunately not often considered, all the aforementioned requirements need to be neatly accommodated to user security and privacy principles. The necessity of privacy and security is crucial for systems that build over sensitive information, and further augmented when data and services are shared by multiple entities in a distributed way. Data ownership, malicious data usage, as well as regulatory and legal policies are important hindrances in the widespread use and acceptance of health and wellness care systems. Therefore, it is of utmost importance to neatly adequate privacy, security, protection and risk management measures to all the processes concerned in a digital health and wellness framework.

## Mining Minds architecture

**Fig. 1:**
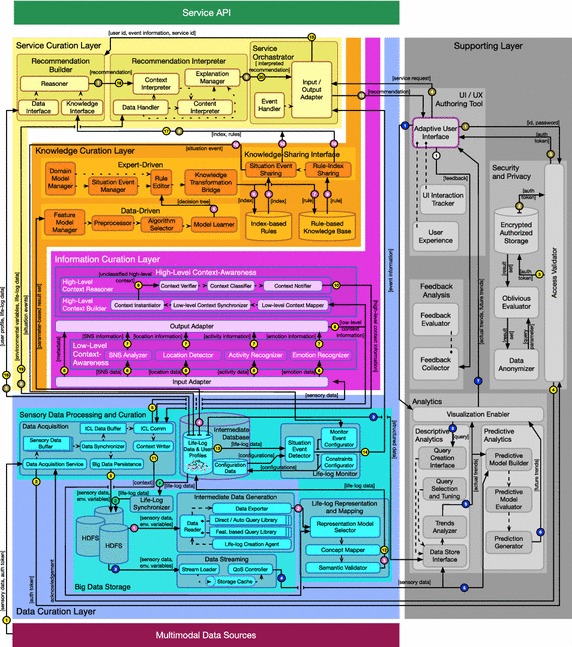
Mining Minds framework architecture and operational diagram

In the light of the aforementioned requirements we present here “Mining Minds”, a novel framework aimed at comprehensively mining human’s daily life data generated from heterogeneous resources for producing personalized health and wellness support. Mining Minds philosophy revolves around the concepts of data, information, knowledge and service curation, which refer to the discovery, processing, adaptation and evolution of both contents and mechanisms for the provision of high quality support services. Motivated by these concepts, a multilayer architecture is particularly devised for Mining Minds—Fig. 1. In a nutshell, the data curation layer (DCL) is in charge of processing and persisting the data obtained from the multimodal data sources (MDS), which abstractly defines the possible sources of user health and wellness data. This includes, but is not limited to, data from social networks, questionnaires, wearable biomedical devices or ambient intelligence systems. The data processed by DCL is primarily used by the information curation layer (ICL) to infer low-level and high-level person-centric information. This information mainly describes the user context and behavior, and, to some extent, their physical, mental and social state. The information extracted by ICL is leveraged by the knowledge curation layer (KCL) to nurture and evolve the health and wellness knowledge primarily created by human experts. Data, information and knowledge are used by the service curation layer (SCL) to create intelligent health and wellness support services, mostly in the form of smart coaching and support recommendations. All the contents and processes are accommodated in terms of security and privacy by the supporting layer (SL), which also provides analysis of user experience, feedback and trends to guarantee the highest personalization.

### Data curation layer

Data curation layer is responsible for acquiring, curating and persisting the data obtained from MDS so it can be processed for higher level understanding. To that end DCL relies on two main modules, Sensory Data Processing and Curation and Big Data Storage. Within the former, Data Acquisition supports the acquisition and synchronization of raw sensory data obtained from diverse sources, both in real-time and offline manner, as generic data streams. Due to the heterogeneous nature of the data, it is acquired asynchronously in real-time and temporarily cached in data buffers. These data buffers are initialized depending upon the number of data sources, i.e., each data source has a data buffer in the Data Acquisition component. All the data buffers are synchronized and communicated to ICL for the determination of the associated low and high-level contexts. In parallel, this synchronized data is stored in Big Data Storage for non-volatile persistence.

Upon receiving the context information determined by ICL, the context instances are curated by the Representation and Mapping component as a time-based log registering the detected human behaviors. This time-based log is termed as user Life-Log or simply Life-Log and persisted in the Intermediate Database for shareability with other layers and applications. The stream of life-log instances is analyzed by a monitoring component called life-log monitor (LLM). The responsibility of the LLM is to perform time-based monitoring of the different attributes and variables hosted in the Life-Log, and support trigger-based mechanisms to notify SCL for the occurrence of an abnormal or special event related to a given user. These abnormal events normally represent risky or unhealthy behaviors and are here defined as “situation events” or “situations” in general. These situations are described through diverse constraints, e.g., age, gender or medical conditions and monitorable variables, e.g., intensity of a particular activity or its duration. Situation events can be generated both statically at design-time and dynamically at run-time upon request from KCL.

The life-log data persisted in the Intermediate Database is regularly synchronized with the Big Data Storage. Big Data Storage also provides read access to raw sensory and life-log data. In case of historic data required by SL for analytics or KCL for data-driven rule generation, Big Data Storage provides queries for data streaming and intermediate data generation. These queries can be customized on request to return specific data based on the attributes selected by KCL and SL. Security and privacy components from SL are further involved in these processes to request authentication and data stream encryption before its persistence or sharing.

### Information curation layer

Information curation layer represents the Mining Minds core for the inference and modeling of the user context [26]. ICL is composed by two main modules, namely, low level context awareness (LLCA) and high level context awareness (HLCA). LLCA is in charge of converting the wide-spectrum of data obtained from the user interaction with the real and cyber-world, into abstract concepts or categories, such as physical activities, emotional states, locations and social patterns. These categories are intelligently combined and processed at HLCA in order to identify more meaningful semantic representations of the user context.

Low level context awareness is composed by four key components, respectively, Activity Recognizer, Emotion Recognizer, Location Detector and SNS Analyzer. The identification of the user physical actions is performed through the Activity Recognizer. This component may build on several sensing modalities as they happen to be available to the user, such as wearable inertial sensors, video and audio. The output of this component corresponds to elementary activity categories such as “sitting” or “walking”. The Emotion Recognizer is defined to infer user emotional states, such as “surprise” or “sadness”, by using video and audio data as well as more sophisticated sources exploring human physiological variations and responses. The user situation is determined by the Location Detector, which essentially builds on the data collected through indoor and outdoor positioning sensors, such as video and GPS, to specify the exact location of the user. The SNS Analyzer is in charge of processing the information generated by the user during their interactions in regular social networks, including posts, mentions, traces and even global social trends, in the form of both text and multimedia data. From here, personal and general interests, conducts and sentiments may be determined. All these components require compatible multimodal sensory data to operate. The provisioning of the necessary data is performed through the Input Adapter, which receives and routes the data curated by DCL to each LLCA component depending on its nature. Once new low-level context categories are identified after the analysis of this data, the Output Adapter serves them to DCL for persistence and to HLCA for further processing.

High level context awareness makes use of two components, namely, High-Level Context Builder and High-Level Context Reasoner, to represent, verify, classify and categorize the user high-level context. The context representation and verification is performed through ontologies, adopted in the past as a unified conceptual backbone for modeling context, while its classification and categorization is done through ontological inference and reasoning. Whenever new information is received from LLCA, a new ontological instance is created by the High-Level Context Builder and categorized into one of the considered high-level contexts by the High-Level Context Reasoner. Thus for example, based on the actual time—e.g., midday; location—e.g., restaurant; and inferred activities—e.g., sitting; this component can determine the precise user context—e.g., lunch.

### Knowledge curation layer

Knowledge curation layer is devised to enable the creation and evolution of both health and wellness knowledge. The knowledge is created either by the domain expert or knowledge engineer by using expert-driven or data-driven approaches. The Expert-Driven module provides a set of rule authoring components to allow specialists to describe in a logical form causes or premises and effects or conclusions, e.g., “if gender is male and age lower than 65 then activity level should be moderate”. The authoring process is further supported through evidence materials and domain vocabularies to confirm the viability of the rules and facilitate their elaboration. The Data-Driven module leverages the contents of the life-log for the automatic generation of rules. To that end, a data broker interface is defined to glean the contents of interest from the data persisted in DCL based on the features or attributes chosen by the expert, e.g., gender, emotional state and activity level. The process is automated by selecting and learning diverse mining models to discover and represent the underlying relationship among the considered health and wellness factors.

In both expert-driven and data-driven cases the generated rules are verified in terms of consistency and validated to avoid potential violations or redundancy with existing rules prior to be stored into the Knowledge Bases. KCL rules are not only persisted in traditional knowledge bases but also indexed according to salient conditions of these rules, also called “causes” or “situations”. These situations refer to particular attributes of the rules than can be monitored by the platform and used for triggering the execution of specific rules. Accordingly, during the rule creation process the expert can select these condition attributes for their particular monitoring at DCL. The categorization of the knowledge bases through these indexes is particularly considered to enhance the performance of the reasoning processes hosted in SCL. In fact, once a situation is detected only its associated rules are shared with SCL through the Knowledge-Sharing Interface upon request of this layer.

The evolution of the knowledge is procured through two main mechanisms. On the one hand, the expert creation process can be considered as a sort of maintenance per se. In that view, rules may be dynamically updated or replaced based on new health and wellness findings from experts. On the other hand, rules can be added, replaced or modified through the data-driven approach while using new life-log contents collected from different users.

### Service curation layer

Service curation layer provides the means to transform the data, information and knowledge curated by DCL, ICL and KCL into actual health and wellness support services. The services are managed by the Service Orchestrator, in charge of attending the potential requests, invoking the necessary services and coordinating the processes involved in the curation of the services. The requests may be of various types, i.e., scheduled on time—e.g, “every day at 8 am”; triggered by direct user queries—e.g., “suggest me an exercise plan for today’s workout”; or based on events—e.g., “user arrives at home”. The last type of request particularly relates to the concept of situation, already described in previous sections. The idea is that the LLM component from DCL triggers SCL whenever a situation event is identified in order to generate a new recommendation for the user.

The services needed to satisfy a given request are invoked from an extensible catalog containing reference and auxiliary services. A major reference service is devised for this architecture for the generation of personalized health and wellness recommendations. This service consists of two parts. First, generalized recommendations are developed by the Recommendation Builder component through reasoning on the user profile and life-log data provided by DCL and the knowledge facilitated by KCL for the specific domain of the service. In the case of handling a request derived from a situation detection the indexed rules hosted by KCL are particularly employed. Second, the recommendations undergo a personalization process through the Recommendation Interpreter in order to deliver the one that best fits the user interests and demands. Through this component all the potential recommendations are filtered based on the user preferences, conditions and possessions, as well as their actual context. Thus, for example, when the objective of the recommendation is to encourage the user to exercise, cycling would be avoided if the user does not own a bike, or a visit to the regular gym omitted in case the person is on a business trip. Similarly, this component can delay the delivery of a given recommendation when it is considered not to be a convenient moment for the user, e.g., if the person is in the middle of a meeting. Prior to be communicated to the user, the recommendation is refined to be easily interpreted by including multimedia contents to increase the interpretability and also incorporating motivational and engagement strategies to foster the user interest and attention.

### Supporting layer

The role of SL is to enrich the overall Mining Minds functionalities through advanced analytics, interactive and personalized UI/UX, implicit and explicit feedback analysis, and adequate privacy and security mechanisms.

The Analytics module is in charge of mining in a multi-dimensional and retrospective manner the data sets collected and curated from multiple users to reveal population health and wellness associations, patterns and trends. These trends may refer to current facts as well as expected or future tendencies. The exploration of present trends is performed through the Descriptive Analytics, which employs statistical techniques to relate explanatory variables of the persisted data. Thus for example, based on the analysis of the inferred people lifestyles, it can be found that there is a growing use of hot beverages among adolescents, which further relates to a dramatic increase of stress patterns. The discovery of potential future facts is carried out by the Predictive Analytics, which develops on the outcomes of the Descriptive Analytics to make forecasts by using regression and machine learning models. Descriptive and predictive analytics contents are organized by the Visualization Enabler, which adjusts the style of the information to be communicated to the users based on their expertise and role.

Evaluating the services supported by Mining Minds requires feedback from the users, which is here powered by the Feedback Analysis component. The sources of feedback may be of a diverse nature, ranging from explicit feedback provided by the user, for example, through questionnaires, to implicit feedback obtained from the user behavioral responses. Analyzing implicit and explicit feedback from the users is motivated by the aspects of functionality, content, and presentation. Functionality-based feedback refers to the findings obtained while comparing, for example, the system recommendations and the behavioral reaction of the user to those recommendations. Content-based feedback measures the user satisfaction with respect to the specific information provided as part of the delivered services. Finally, presentation-based feedback measures the human-computer interaction with respect to the user interface (UI), which is of particular utility to understand the user experience (UX). All these types of feedback are devised to help assessing the level of interest and adherence of users to the services provided through Mining Minds as well as to evolve and maintain the internal contents and processes handled by the platform.

Considering user preferences, habits or mood, the UI/UX module enables the end-user applications interface to be adapted accordingly. This adaptation is needed to adjust the human-computer interaction experience with respect to font size, theme, or audio levels, among other characteristics. Two main components are involved in this process. First, the UI Interaction Tracker collects the data from the interaction between the person and the application to analyze the user’s ability to understand and use the system, e.g., the readability of the contents or the perceptibility of the controls. Then, the UX component measures the satisfaction level based on the analysis of the collected data. The immediate result is a dynamic adaptation of the UI based on the measurements extracted from the evaluation of the UX.

Given the sensitivity of the collected user data, privacy and security need to be assured and exhibited, not only for storage, but also during the processing and delivery of services. To that end, state-of-the-art cryptographic primitives along with indigenous protocols are considered. For secure storage, the AES standard is particularly used, whereas for oblivious processing, homomorphic encryption and private matching is used. Considering the intensive data flow between end-user applications and Mining Minds, data randomization techniques are used to ensure a high entropy for minimal leakage of information. An authorized model ensures the legitimate disclosure of personal data and services with users. Slow processing of information is a common byproduct of the encryption; thus, to assist partial swiftness to Mining Minds, sensitive and non-sensitive information is decoupled where required. Anonymization procedures are also considered to enable the use of the collected and mined users data by third party agents, e.g., for research purposes.

## Mining Minds implementation

An initial implementation of the proposed framework particularly oriented to promote healthy lifestyles and physical activity management is described here. Mining Minds is a distributed platform where the cloud environment plays a key role for supporting both persistence and limitless computational power. The Mining Minds implementation has been deployed over a hybrid cloud combining Microsoft Azure public cloud environment [27] and a Xen private cloud [28] for the big data storage, which runs over Hadoop File System with MapReduce [29]. For better scalability and performance each layer is deployed over a separate virtual instance on Microsoft Azure. DCL, ICL, KCL and SCL are hosted on standard Microsoft Azure instances with Windows Server 2012 R2 as guest operating system [30], while SL functionalities partake of the others. The cloud-based deployment of layers allows the encapsulation of their responsibilities as well as the re-usability of their features through an inter-layer communication. This communication is implemented by establishing service contracts among the layers, which communicate by means of RESTful web services [31] and high performance sockets [32]. The communication between MDS and DCL is implemented through sockets given its real-time nature. Similarly, a high performance socket-based implementation is particularly used for DCL-ICL communication for the transference of sensory data and context determination in real-time, and communication between DCL and the big data storage on private cloud. The most important service contracts with remaining layers are supported by DCL RESTful web services, which serve a data model with the structure of the Intermediate Database, here hosted by Microsoft SQL Server [33]. This data model is shared among the layers as an object model of service contract. The required data and information is populated by DCL and provided as responses to the upper layers.

To support active lifestyle services in this version ICL only implements the Activity Recognizer. This component consists of various steps that mainly combine signal processing and machine learning techniques to define a specific human activity recognition model capable of distinguishing among various commonplace activities [34]. The main input of this model is body motion data, namely, acceleration, which can be broadly obtained from smartphones and wearable inertial sensors. Acceleration is preferentially used here since it is the most prevalent sensor modality in standard activity recognition approaches [35]. A non-overlapping sliding window of three seconds is used for the data segmentation [36], and time and frequency features extracted for their discrimination potential [37]. The implemented model combines Support Vector Machines and Gaussian Mixture Models for the classification process, which have been demonstrated of particular utility in this domain [38, 39]. The developed Activity Recognizer further supports three operation modes depending on the available data registered from the user. Specifically, a hierarchical approach is developed so that the model can determine the user activity based only on the inertial data collected through the smartphone, smartwatch or a combination of smartphone and smartwatch data if available.

Health and wellness knowledge is defined by medical experts and hosted in the Knowledge Bases of KCL. To that end, a simple rule authoring tool [40] is considered for the rule creation. Evidences and domain vocabularies are particularized to the definition of physical management and activity promotion plans [41]. SCL processes the contents generated by DCL, ICL and KCL for the generation of personalized physical activity recommendations. After a request is processed by the service orchestrator, generalized recommendations are produced by applying rule-based reasoning [42] on the existing knowledge and user data. User health and wellness data is transformed into a proper input query by using auxiliary services hosted in the service catalog. Similarly, auxiliary services are implemented for user goal discovery, e.g., ideal weight and calories to be burned per day [43]. During the reasoning, the interpreter analyzes each rule in the knowledge bases and fires the appropriate rules using a forward chaining procedure [44]. Recommendations are personalized by using content-based filtration techniques [45] employing user-centric information such as activity level and preferred physical activities.

Security and privacy components of SL are distributed among the different layers. Encryption techniques are employed to withstand any compromise on data storage facility or its unauthorized access, as well as to make health-related data processing and evaluation HIPAA compliant. Concretely, AES [46], private matching [47] and multidimensional anonymization [48] have been chosen to support the encryption and control access. Moreover, since the systems are deployed on public clouds, processing over direct encryption without losing accuracy is required. The indigenously proposed system of oblivious term matching [49] is considered here to that end.

## Health and wellness promotion services

**Fig. 2:**
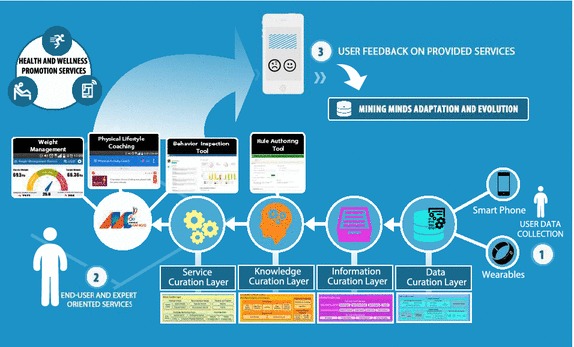
Mining Minds health and wellness service scenario

Various exemplary applications and tools have been developed to showcase some of the potential health and wellness services supported by Mining Minds—Fig. 2. Personalized weight management is procured through an application that promotes activity routines customized to the user characteristics and preferences in order to attain a healthy weight. The app further provides the person with valuable information regarding their physical behavior, energy expenditure and weight loss patterns. Behavior change and healthy lifestyle promotion is intended through a personal coaching application which delivers action recommendations and educational facts upon detection of unhealthy physical conducts. Conversely to other digital health and wellness systems and platforms, Mining Minds is not only devised to support regular users or patients but also specialists. Medical experts are facilitated with a comprehensive tool to inspect users behavior, engagement and satisfaction in a continuous and retrospective manner. Apart from diverse statistics reporting personal goals, achievements and physical activity patterns, the tool allows the specialist to check the specific information and recommendations delivered by the platform to each particular user. Finally, an intuitive rule authoring tool has also been developed to enable the creation and management of the health and wellness knowledge exploited by Mining Minds. The main features and utilities of these applications and tools are described next.

### Personalized weight management app

**Fig. 3:**
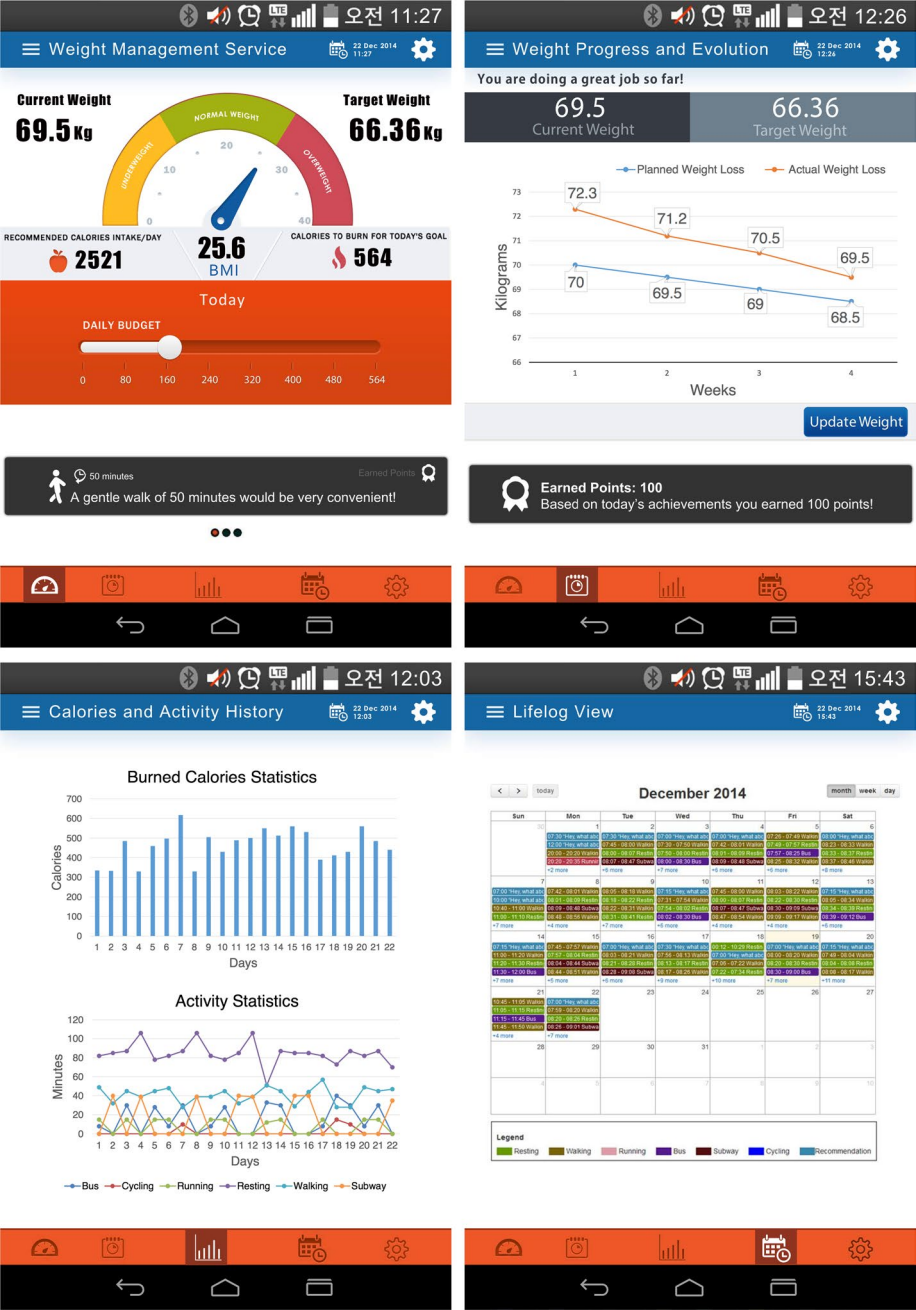
Personalized weight management app

A poor estimation of calories and activities as well as an unrealistic definition of milestones represent two of the most common reasons for failure in most weight loss programs. Accordingly, the main objective of this service is to empower people in the control of their weight through a continuous track of exercise and energy consumption and a personalized physical routine promotion to achieve the daily expenditure goals. Users are initially requested to sign up into the application by entering their personal information such as demographics—age, gender, weight and height, preferences in terms of activities and exercise level—sedentary, moderate or intense. All this information is securely stored and processed by the Mining Minds platform to calculate the user physical state, ideal weight, as well as the calories to be burned every day, all displayed for simple access on the app main dashboard—Fig. 3, top-left. The amount of calories burned by the user in the present day is also displayed on this view. This value is estimated by the platform by analyzing the user activity patterns. To determine these patterns, Mining Minds elaborates on the acceleration data measured by the user smartphone, which is timely streamed through WiFi or 4G to the platform. To promote the user activity to achieve the daily calorie goal, exercise recommendations are given in an easy-to-understand manner. The recommendations contain precise indications on the duration of the activity and its execution style as well as motivational statements for encouraging the user. The recommended activities, their duration and intensity are personalized to each individual based on their profile. The evolution of the user's actual weight with respect to the planned one is presented in a different frame—Fig. 3, top-right. Here the user can easily self-report their current weight upon timely request of the platform. Other supportive features of the application provide the user with statistical analysis of burned calories and activity patterns —Fig. 3, bottom-left, and a calendar view of the user comportment—Fig. 3, bottom-right, specifically devised to support users in their self-monitoring and control.

### Physical lifestyle coaching app

**Fig. 4:**
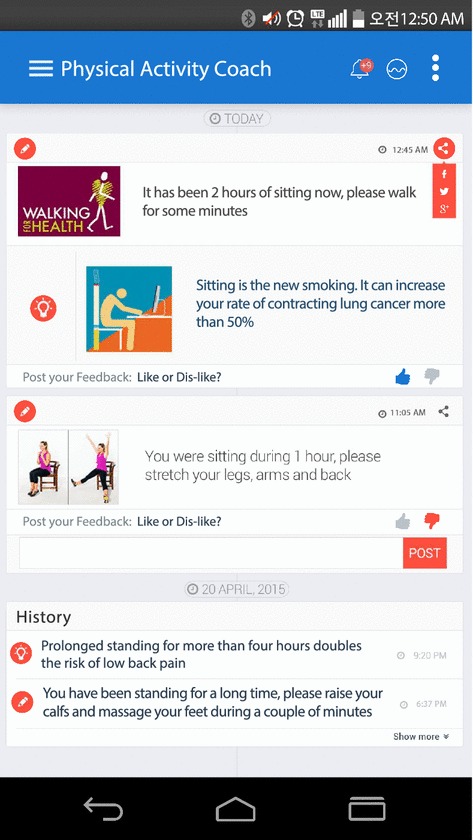
Physical lifestyle coaching app

Behavior change and healthy lifestyle promotion constitute central objectives in public health interventions. The service defined here explores sophisticated coaching mechanisms to raise people’s health awareness while inducing wholesome activity habits, changing unhealthy routines and educating on healthier physical lifestyles. To that end the developed application continuously captures the user’s body motion data registered through the inertial sensors of the smartwatch and smartphone. The data is then streamed to Mining Minds which processes it to infer the user's behavior and determine potential risk or unhealthy situations. After an unhealthy behavior is detected, e.g., “one hour of continuous sitting”, the platform automatically generates a personalized physical recommendation or healthy educational fact, e.g., “stretch your legs, arms and back”. Recommendations and facts are conveniently delivered according to the user context and availability, and displayed on the application main screen in a timeline view—Fig. 4. Both recommendations and facts are also accompanied by multimedia contents—video, images and audio—to instruct the user on how to follow them as well as to attract and increase their interest and understanding. Moreover, users can value the delivered recommendations and facts according to their experience—“likes”/“dislikes”— and also provide comments on them—e.g., “I cannot carry out the recommended stretching exercises” or “My back hurts when I bend my waist”. This information constitutes a key source of feedback for experts and Mining Minds itself to realize the comprehensiveness, applicability and impact of the services delivered by the platform.

### Behavior inspection tool

**Fig. 5:**
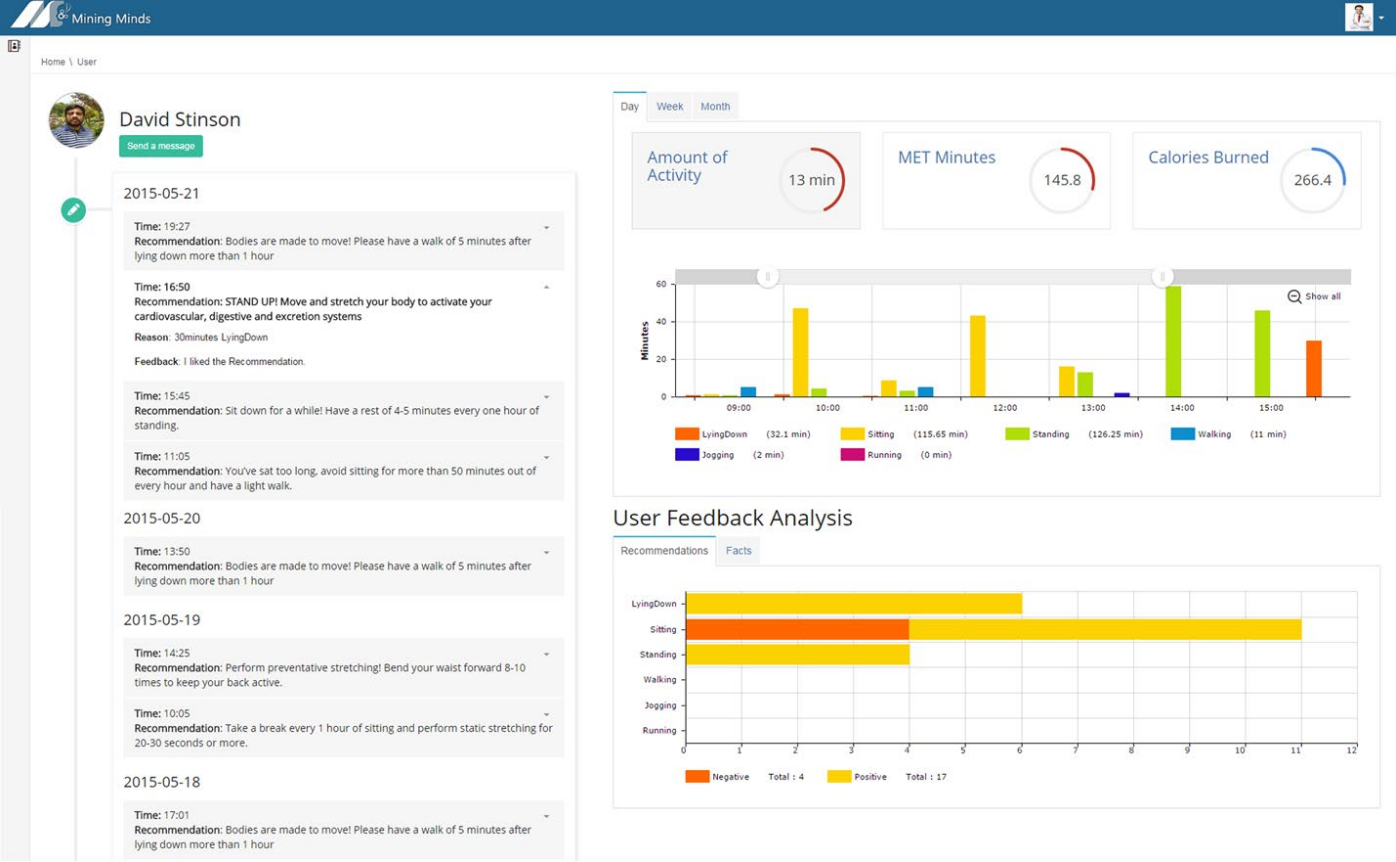
Behavior inspection tool

Intelligent monitoring and smart coaching mechanisms are not planned to replace the role of specialists but rather complement it. In fact, the idea is that not only patients but also medical experts can benefit from the data, information, knowledge and services handled by Mining Minds. The expert inspection tool developed here is particularly devised to facilitate and expedite the task of health and wellness counseling specialists. The tool— Fig. 5—presents in an intuitive yet comprehensive fashion some of the most prominent user-centric information managed by the platform. On the left side of this expert view the specialist can check the recommendations and facts delivered by Mining Minds to the user, the reason behind these suggestions as well as the feedback provided on them. On the right side, diverse sort of analytics describing the physical achievements of the user, their behavioral patterns and their rating of recommendations and facts are shown. Energy expenditure achievements and physical activity patterns are displayed in a daily, weekly and monthly basis, thus providing the expert with a detailed view of the user past and present status. The user feedback analytics is of particular interest to help experts identify what kind of recommendations and facts are more positively valued and which ones may not be accepted. The tool is also incorporated with a feature that allows the specialist to directly communicate with the user through the developed apps by sending comments in the form of notifications. Through this tool experts can in principle deal with more users while reducing the time required for the assessment of their progresses and evolution.

### Rule authoring tool

**Fig. 6:**
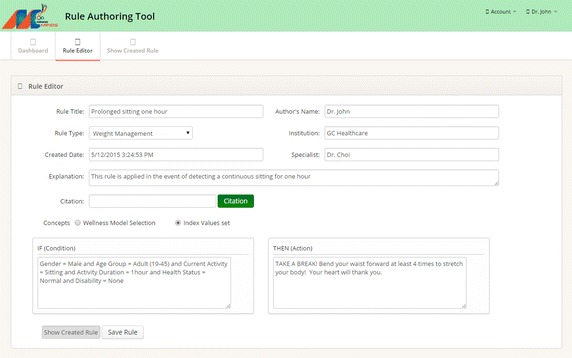
Rule authoring tool

Health and wellness experts are not only consumers of the services supported by Mining Minds but also content producers. The creation and management of Mining Minds health and wellness knowledge is handled by the specialists through an advanced rule authoring tool—Fig. 6. This rule authoring tool is an adapted version of a prior one first introduced in [40]. The rule authoring tool provides domain experts with an easy to use dashboard to manage the existing rules, thus making possible their addition, update or deletion. An intuitive environment is provided for the creation of new rules and associated meta-information. The rule authoring tool incorporates a sophisticated physical activity wellness model which incorporates multiple domain concepts and vocabularies facilitating the rule creation task. The tool is also equipped with intelligent code completion technology to expedite the rule creation process and reduce the chance of errors. After the rule is created, the expert can simply save it, thus making it available for its use in Mining Minds.

## Evaluation and discussion

A preliminary evaluation of the implemented version of the platform and services is performed here. An important asset of the platform refers to the curation and persistence of sensory data by DCL. Most health applications delete sensory data after processing it; however, persisting this information is of worth for generating datasets that can be used to evolve the knowledge models or learn new ones. To benchmark DCL capabilities, the accuracy and performance of the platform in the collection, processing and storage of the sensory data is measured. To that end, continuous data service calls over the period of 24 h are generated and evaluated. The accuracy is measured by the rate of missing data packets, here summarized in Table 1. The results show a very low error, 0.06 % in average, which means that practically all the sensory data sent to the platform is safely processed. The performance, presented in Table 2, measures the capacity of the system to store the data packets into the platform storage. The stress test shows a high consistency with the increasing usage of the system, which is capable of writing 2.2 requests or packets per second in average, each one composed by 7800 records of sensory data.

**Table 1:** Accuracy of the data curation process

No. of service calls	No. of missed data packets	Packet loss error (%)
30,000	6	0.02
60,000	22	0.04
90,000	39	0.04
120,000	55	0.05
150,000	96	0.06
180,000	308	0.17
Average		0.06

**Table 2:** Performance of the data persistence process for different operation runs

Run duration (h)	2	4	6	8	10	12	14	16	18	20	22	24
Performance (avg data writes/s)	2.20	2.21	2.20	2.20	2.10	2.21	2.21	2.21	2.20	2.20	2.20	2.20

The capability of ICL for inferring user activities presents important advantages with respect to other wellness systems, which frequently rely on simple step counting for activity tracking. For example, it permits to derive more precisely the user energy expenditure based on the cost of each performed activity, specially for those that do not entail any ambulation. To evaluate the potential of the implemented activity recognition model ten volunteers aged from 23 to 37 years were requested to perform the supported activities, namely, “walking”, “jogging”, “running”, “stretching”, “sweeping”, “eating”, “sitting”, “standing” and “lying down”. The performance of the model is evaluated by comparing both actual and detected activities for three different scenarios defined upon the available sensing technology, respectively, smartphone—Table 3, smartwatch—Table 4 and both devices—Table 5. The results prove notable recognition capabilities in general, yielding an overall F-score of 0.93 for the case in which only the smartphone is used for registering the user’s body motion, 0.92 when the smartwatch is solely employed, and 0.95 when both devices are used.

**Table 3:** Activity recognition performance when operating on the smartphone data

Activity	SE	SP	PPV	NPV	F-score
Eating	0.87	0.99	0.86	0.99	0.86
Running	0.97	1.00	1.00	1.00	0.99
Sitting	0.94	0.98	0.93	0.98	0.94
Standing	0.88	0.99	0.95	0.98	0.91
Walking	0.99	0.99	0.98	1.00	0.99
Jogging	0.99	1.00	0.98	1.00	0.99
Stretching	0.96	0.99	0.91	1.00	0.93
Sweeping	0.93	0.99	0.90	0.99	0.91
Lying down	0.84	1.00	0.92	0.99	0.88

Each metric correspond to * SE* sensitivity,* SP* specificity, *PPV* positive predictive value,* NPV*, negative predictive value and F-score

**Table 4:** Activity recognition performance when operating on the smartwatch data

Activity	SE	SP	PPV	NPV	F-score
Eating	0.82	0.99	0.86	0.99	0.84
Running	0.96	1.00	0.94	1.00	0.95
Sitting	0.93	0.94	0.85	0.97	0.89
Standing	0.85	0.99	0.94	0.97	0.90
Walking	0.96	0.98	0.95	0.98	0.96
Jogging	0.92	1.00	0.97	1.00	0.95
Stretching	0.93	1.00	0.97	0.99	0.95
Sweeping	0.95	1.00	0.99	1.00	0.97
Lying down	0.85	1.00	0.93	0.99	0.89

Each metric correspond to* SE* sensitivity, *SP* specificity,* PPV* positive predictive value,* NPV* negative predictive value and F-score

An initial user-centric analysis is also performed in terms of adherence to the provided recommendations. Ten independent volunteers between the ages of 26 and 38 years were asked to use the developed applications during a couple of weeks to measure the response time to the recommendations generated by the platform. This time accounts for the period elapsed since the user receives a recommendation and follows it. The average number of recommendations per day were 9, ranging from 5 to 14. The subjects response time varied from 1 min to 1 h, with average values shown in Table 6. These results may give some hints on the interest shown in the use of these services, although further analysis, including more subjects and longer time spans, is required to obtain solid conclusions.

**Table 5:** Activity recognition performance when operating on both smartphone and smartwatch data

Activity	SE	SP	PPV	NPV	F-score
Eating	0.89	1.00	0.88	1.00	0.88
Running	0.97	1.00	0.99	1.00	0.98
Sitting	0.95	0.98	0.94	0.98	0.95
Standing	0.91	0.99	0.95	0.98	0.93
Walking	0.99	0.99	0.98	1.00	0.99
Jogging	0.98	1.00	0.98	1.00	0.98
Stretching	0.97	0.99	0.92	1.00	0.94
Sweeping	0.94	1.00	0.94	1.00	0.94
Lying down	0.90	1.00	0.93	1.00	0.92

Each metric correspond to* SE* sensitivity, *SP* specificity,* PPV* positive predictive value,* NPV* negative predictive value and F-score

**Table 6:** Average user response time (in minutes) to recommendations

User	1	2	3	4	5	6	7	8	9	10
Avg response time	24.47	34.44	3.42	5.38	40.44	7.21	28.29	13.99	8.56	36.84

Finally, the effectiveness and usability of the developed expert tools is also assessed. To that end, different aspects of the tools were evaluated by six medical experts—two nutritionists, two fitness instructors and two nurses—from an independent health and wellness counseling company. The experts were instructed on how to use the tools and then provided with a set of questionnaires to evaluate their look and feel, interface layout complexity, time required to access a given resource or create a new rule and the understandability and correctness of the concepts and contents facilitated by these tools. The results of the evaluation prove a satisfaction level of 7.5 out of 10 in average. The aspects that were more highly rated correspond to the usefulness, organization and simple access to the displayed health and wellness information. For the behavior inspection tool the experts particularly valued having a user-centric description of the behavioral patterns plus the possibility of identifying the acceptability of the delivered recommendations through the feedback analysis report. For the rule authoring tool the specialists especially considered the benefits provided by the health and wellness models during the rule creation process, although they showed some concerns regarding the amount of time required to write a given rule. All the positive and negative feedback obtained through all these evaluations is being considered at the moment for evolution and improvement of the developed services.

It is worth noting that all the developed apps and tools have been designed as end-user interfaces to the contents and services curated by Mining Minds, thus presenting important advantages for the customers, such as an effective reduction of the resources consumption—mainly in terms of storage, computation and battery, no need of regular updates of the client application, shareability of contents among diverse systems and applications, as well as a more dynamic and interactive experience. Despite these important advantages, there are some limitations that need especial consideration. Mining Minds builds on the assumption of having most mobile devices and systems of the Internet of Things fully and seamlessly connected in the near future. However, this condition is currently not always satisfied; therefore, the applications may require to support temporary local storage and offline data transmission to overcome potential network disconnections. Another open issue refers to the cost of the communication between the apps and the platform. Applications such as the ones presented here operate over WiFi and 4G interfaces. While the use of WiFi presents no economic burden, some users could be concerned about using their data plans when huge amounts of data need to be transferred. For example, the developed user applications transmit around 500kB/min to communicate the sensory data to the platform, which translates into approximately 30GB/month when used non-stop. With the advent of 5G communications, flat-rate data plans are expected to become commonplace, thus helping to reduce the possible burden for the end-user. In either case, the utilization of compressed sensing techniques [50] is particularly envisioned for the future to make the data transmission more efficient. These mechanisms and other sophisticated strategies are also worth considering to reduce battery consumption, for example, by interrupting the transmission of sensory data during periods of user inactivity.

## Conclusions

This work has presented Mining Minds, a novel digital framework for personalized healthcare and wellness support. The framework has been neatly designed taking into account crucial requirements of digital health and wellness systems. This work has also described a unique architecture defined to provide the necessary functionality to enable curation and mining of data, information, knowledge and services for personalized health and wellness support. An initial realization of the key architectural components as well as various exemplary applications and tools showcasing some of the benefits provided by Mining Minds have also been presented and evaluated. The work is ongoing to complete the implementation of the devised architecture with new additional components, as well as to evaluate its services on a large-scale testbed.

## Consent

Written informed consent was obtained from the participants for publication of this case report and any accompanying images.
